# Current and future strategies to monitor and manage coagulation in ECMO patients

**DOI:** 10.1186/s12959-023-00452-z

**Published:** 2023-01-26

**Authors:** Saeedreza Zeibi Shirejini, Josie Carberry, Zoe K. McQuilten, Aidan J. C. Burrell, Shaun D. Gregory, Christoph E. Hagemeyer

**Affiliations:** 1 NanoBiotechnology Laboratory, Central Clinical School, Australian Centre for Blood Diseases, Monash University, Melbourne, VIC Australia; 2 Cardiorespiratory Engineering and Technology Laboratory, Department of Mechanical and Aerospace Engineering, Monash University, Clayton, VIC Australia; 3 Department of Mechanical and Aerospace Engineering, Monash University, Clayton, VIC Australia; 4 Transfusion Research Unit, School of Public Health and Preventive Medicine, Monash University, and Department of Clinical Haematology, Monash Health, Melbourne, VIC Australia; 5 School of Medicine, Nursing, and Health Sciences, Clayton and Intensive Care Unit, Monash University, Alfred Hospital, Melbourne, VIC Australia; 6 Department of Epidemiology and Preventative Medicine, School of Public Health, Monash University, Melbourne, VIC Australia

**Keywords:** Anticoagulation, Extracorporeal Membrane Oxygenation, Monitoring Techniques, Thrombosis, Bleeding, Respiratory Failure, Heparin-Induced Thrombocytopenia (HIT)

## Abstract

Extracorporeal membrane oxygenation (ECMO) can provide life-saving support for critically ill patients suffering severe respiratory and/or cardiac failure. However, thrombosis and bleeding remain common and complex problems to manage. Key causes of thrombosis in ECMO patients include blood contact to pro-thrombotic and non-physiological surfaces, as well as high shearing forces in the pump and membrane oxygenator. On the other hand, adverse effects of anticoagulant, thrombocytopenia, platelet dysfunction, acquired von Willebrand syndrome, and hyperfibrinolysis are all established as causes of bleeding. Finding safe and effective anticoagulants that balance thrombosis and bleeding risk remains challenging. This review highlights commonly used anticoagulants in ECMO, including their mechanism of action, monitoring methods, strengths and limitations. It further elaborates on existing anticoagulant monitoring strategies, indicating their target range, benefits and drawbacks. Finally, it introduces several highly novel approaches to real-time anticoagulation monitoring methods including sound, optical, fluorescent, and electrical measurement as well as their working principles and future directions for research.

## Introduction

**Fig. 1:**
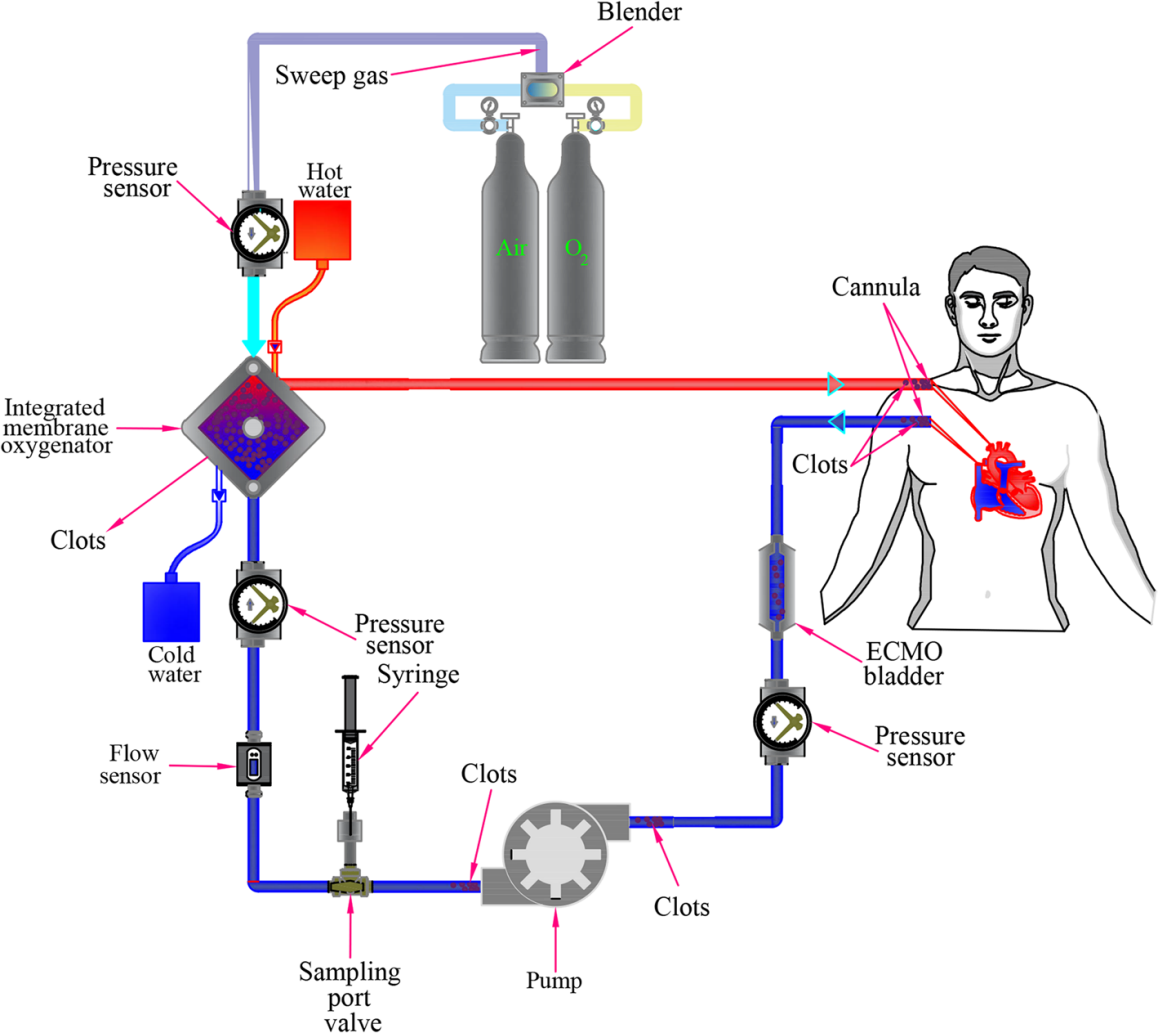
Schematic of ECMO setup indicating the most likely clot formation sites

Extracorporeal membrane oxygenation (ECMO) is a treatment option for patients with advanced cardiorespiratory failure and can be utilized in both children and adults [1]. ECMO is a life-saving heart–lung machine that delivers oxygen to patients with refractory severe respiratory or cardiac failure as they await a transplant or recover from a serious illness for a few days or weeks [2]. In an ECMO circuit, the deoxygenated blood drained from the venous circulation is pumped to the oxygenator via a pump to exchange carbon dioxide with oxygen. The blood is then returned to either the venous (VV-ECMO) or arterial (VA-ECMO) circulation [3]. The ECMO circuit components (Fig. 1) include a pump, membrane oxygenator, heat exchanger, venous cannula, arterial or venous infusion canula, tubing, and connectors [4].

The most frequent complications and one of the common causes of death for patients on ECMO are thrombus formation and bleeding [5, 6]. The risk of bleeding is related to patient-specific and treatment-related factors associated with ECMO. According to Virchow's triad, which explains the thrombosis etiologies as hypercoagulability, vessel wall injury, and blood flow stasis, the ECMO circuit contains non-biological surfaces, regions of very high shear stress and regions of prolonged blood residence time, all of which act to promote thrombus formation at a level requiring systemic anticoagulation, which in turn increases bleeding complications [7].

ECMO patients are frequently critically ill, increasing the risk of bleeding complications [8]. The bleeding rate during ECMO is 20.8–39.6% [6, 9] with the cannula site (13.2%), gastrointestinal tract (5.5%), lungs (6.1%), and central nervous system (3.9%) being the most prevalent sites [10]. ECMO patients are also at risk of thrombosis complications, including ischemic stroke, right ventricular thrombus [11], left ventricular thrombus [12], and pulmonary embolism [13]. The rate of thrombosis formation in patients undergoing ECMO is 10–46.1% of patients depending on the circuit type and age of the patient in various centres [14]. Finding the balance between bleeding and thrombosis necessitates continuous monitoring of various parameters including coagulation factors, fibrinogen, and platelets. In this review, conventional and recently developed anticoagulants used in ECMO are presented, including their mechanism of action, monitoring methods, strengths and limitations. It expands on existing anticoagulant monitoring systems, indicating their target range, advantages, and disadvantages. It also introduces various unique real-time coagulation monitoring techniques, as well as their operating principles and future research prospects.

### Anticoagulation during extracorporeal circulation

**Fig. 2:**
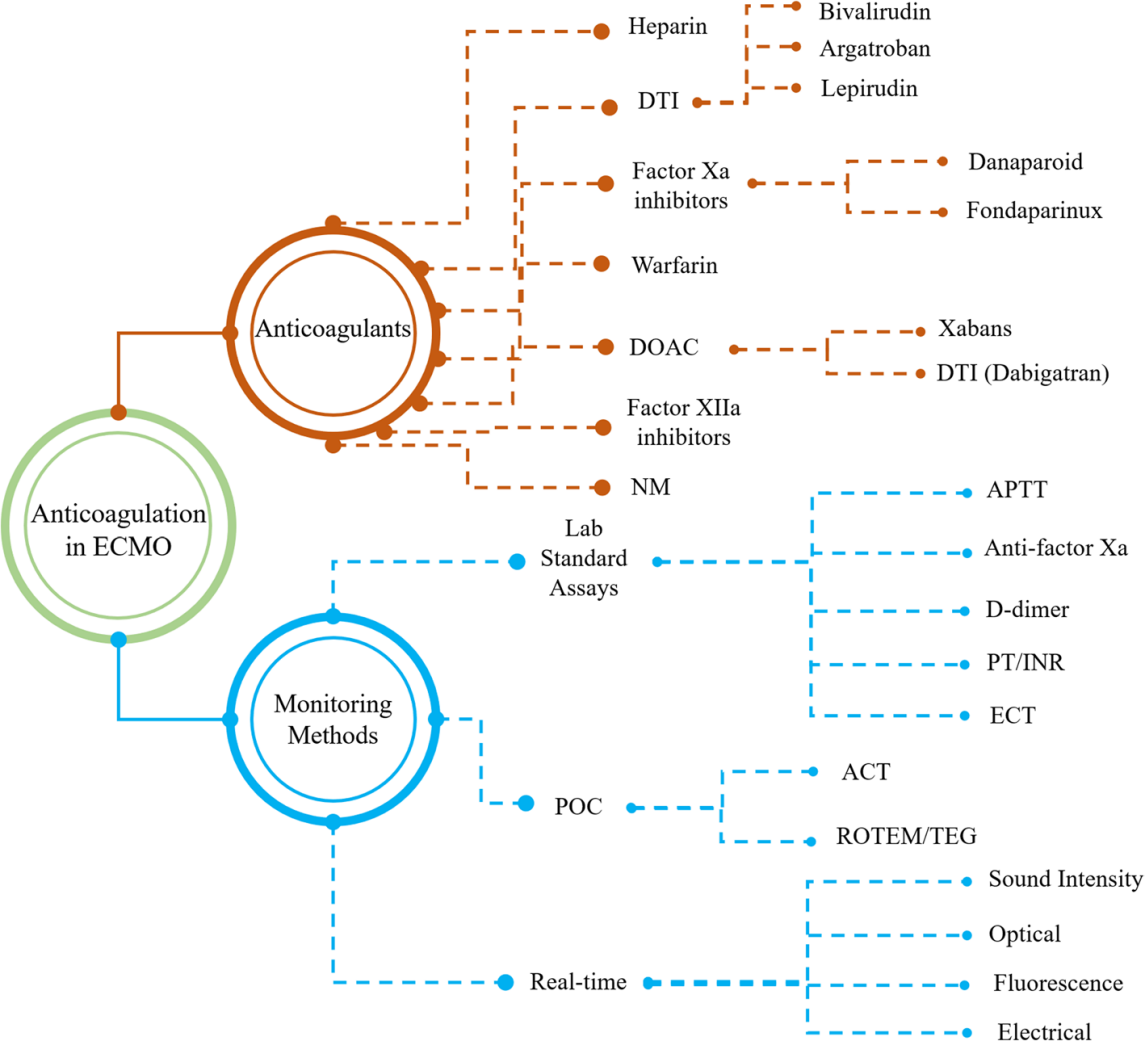
Various anticoagulants used in ECMO devices and their monitoring methods (AT: Antithrombin; DTI: Direct Thrombin Inhibitor; DOAC: Direct Oral Anticoagulant; NM: Nafamostat mesylate; FBC: Full Blood Count; aPTT: Partial Thromboplastin Time; PT/INR: Prothrombin time/international normalized ratio; ECT: Ecarin Clotting Time; POC: Point-of-Care; ACT: Activated Clotting Time; ROTEM: Rotational Thromboelastometry; TEG: Thromboelastography)

Firstly, anticoagulants utilized in ECMO are summarized, including their benefits and limitations to provide a comprehensive viewpoint for future research. In addition, this review takes a fresh view on anticoagulant monitoring strategies by classifying them according to their purpose (Fig. 2).

### Anticoagulant agents

Anticoagulants commonly used for anticoagulation in ECMO patients include heparin, direct thrombin inhibitors (DTIs) (bivalirudin, argatroban, lepirudin), factor Xa inhibitors (danaparoid and fondaparinux), direct oral anticoagulants (DOAC) (DTI (dabigatran), Xabans (rivaroxaban, apixaban, darexaban, edoxaban, and betrixaban)), factor-XIIa inhibitors, nafamostat mesylate (NM), and warfarin so that heparin and DTIs are the most prevalent anticoagulants used in ECMO patients. Although heparin is the most commonly used in clinical applications, heparin resistance is a major concern in ECMO. It is defined as a situation where the ability of heparin to inhibit thrombin (factor IIa) and fibrin formation is reduced such that the correlation between dose and response is lost and increasing the heparin dosage will not result in the desired anticoagulation effect [7, 15]. Moreover, its usage is associated with the rare but life threatening immune-mediated disorder Heparin-induced thrombocytopenia (HIT), specified by thrombocytopenia and a paradoxical prothrombotic state in heparin treatment [16]. Alternative anticoagulants, on the other hand, offer excellent potential for usage in HIT patients, but present some other challenges. The working mechanism, advantages, disadvantages, and other aspects of different anticoagulants used during ECMO support including heparin and its alternatives are provided in Table 1.

**Table 1:** Anticoagulant Agents

Anticoagulant	Mechanism	Typical dosing	Monitoring	Half-life	Elimination	Reversal agent (Antidote)	Administration	Advantages	Disadvantages	Ref
Heparin	Enhance the AT activity to inactivate thrombin; Inhibiting anti-FXa and AT	A bolus initial dose of 40–100 U/kg followed by a continuous infusion of 10–50 U/kg/hr, targeted to an aPTT or Anti-Xa level	Anti-factor Xa, ACT, aPTT, ROTEM/TEG, AT assay	90 min	Renal and hepatic	Protamine	Intravenous (IV)	● Well known mechanism of action ● Easy to monitor ● Easy titration ● Ease of reversibility with protamine ● Inexpensive	● Non-linear dose–response relationship ● HIT induction ● Heparin resistance ● Depends on the level of AT	[61]
Bivalirudin	Reversible direct thrombin inhibitor	It can be used with or without a bolus; A bolus initial dose of 0–0.75 mg/kg and a maintenance dose of between 0.025 and 0.5 mg/kg/h	Plasma level of drug, ECT, aPTT, diluted thrombin time (dTT), ACT	25 min	Renal (20%) and hepatic (80%)	None	IV	● No HIT induction ● Short half-life ● Independent of AT levels	● Bivalirudin resistance requiring dose escalation ● Interferes with fibrin production, platelet aggregation, and factor XII activation ● Prolonged half-life and drug accumulation in renal failure	[62]
Argatroban	Reversible direct thrombin inhibitor	Typically, no bolus loading is required (However, some centres use 100–200 $\mathrm{\mu g}/\mathrm{kg}$*)* A maintenance infusion of rate between 0.1 and 2 $\mathrm{\mu g}/\mathrm{kg}/\mathrm{min}$	aPTT, diluted thrombin time, ACT, ECT, ROTEM/TEG	39 to 51 min; depending on hepatic function	Hepatic	None	IV	● No HIT induction ● Fewer transfusions than heparin ● Independent of AT levels	● Increased dose required to maintain therapeutic range over time ● Less familiar to most clinicians ● Long onset of action ● Interferes with fibrin production, platelet aggregation and factor XII activation ● Prolongs the aPTT, ACT, PT/INR results	[63]
Danaparoid	Factor Xa inhibitor	200–300 U/hr	Anti‐factor Xa assay	19‐25 h	Renal	None	IV and subcutaneous	● Recommended for HIT situation ● Some anti-thrombin activities	● Has been unavailable for several years due to manufacturing issues	[64]
Fondaparinux	Factor Xa inhibitor	2.5–10 mg/day	Anti‐factor Xa assay	17–21 h	Renal	None	IV and subcutaneous	● High level of activity ● Recommended for HIT situation ● Its activity is 20 times greater than the danaparoid	● Does not inhibit thrombin ● Major bleeding in HIT patients ● Restricted usage in renal dysfunction situation ● Not for use while actively undergoing ECMO	[65]
Nafamostat mesylate	Inhibits many procoagulant factors including thrombin, factors XIIa, Xa, and antifibrinolytic and anti-platelet action	0.26–0.93 mg/kg/hr	ACT, aPTT	8 min	Renal	None	IV	● Short half-life	● Limited published data on its safety and efficacy	[66]
Warfarin	Inhibits vitamin K-dependent clotting factors (II, VII, IX, X)	Initial dose of 10 mg and maintenance dosage of 1–2 mg per day	INR	20–60 h	Mainly hepatic and minor enzymatic	Vitamin K, prothrombin complex concentrates (PCC) and fresh frozen plasma (FFP)	IV or orally	● Long-term anticoagulation after ECMO ● Easy administration ● Reversibility	● Not for use while actively undergoing ECMO ● Not a good choice for acute HIT situation ● High drug interactions and dose adjustment required	[67]
Dabigatran	Reversible direct thrombin inhibitor	75–150 mg per day	ACT, aPTT, ECT and thrombin time (TT)	12–17 h, up to 34 h in renal failure	Renal	Idarucizumab (Praxbind)	Oral	● Does not require frequent laboratory monitoring due to its low drug–drug and drug–food interactions ● Reversibility	● Induces dyspepsia ● High cost ● Limited published data on its safety and efficacy ● Limited application in ECMO due to long onset of action	[68]
Rivaroxaban	Factor Xa inhibitor	15–20 mg per day	Prothrombin time, Anti‐factor Xa assay	5–9 h in healthy young people and 11 to 13 h in elderly people	Both renal and hepatic	Andexanet alfa	Oral	● Does not require frequent laboratory monitoring ● Reversibility	● Limited information about using DOACs in ECMO patients due to long onset of action and prolonged half-life	[69]

*AT* Antithrombin, *TT* Thrombin Time, *dTT* Diluted thrombin Time, *DTI* Direct Thrombin Inhibitor, *DOAC* Direct Oral Anticoagulant, *NM* Nafamostat mesylate, *IV* Intravenous, *HIT* Heparin-Induced Thrombocytopenia, *FBC* Full Blood Count, *aPTT* Partial Thromboplastin Time, *PT/INR* Prothrombin time/international normalized ratio, *ECT* Ecarin Clotting Time, *POC* Point-of-Care, *ACT* Activated Clotting Time, *ROTEM* Rotational Thromboelastometry, *TEG* Thromboelastography)

### Heparin

UFH is the most frequently used anticoagulant in patients undergoing ECMO due to its advantages including its low cost, titratability, and easy reversibility by protamine. Heparin inhibits thrombin by binding to antithrombin (AT). AT has low anticoagulation activity but when conjugated with heparin, its anticoagulation activity increases 1000–2000-fold [17]. Heparin resistance is the main concern in ECMO, which is defined as a situation where the heparin ability to inhibit thrombin and fibrin formation is reduced so that heparin dosage response is not correlated with the injected amount of heparin and increasing the heparin dosage will not result in the desired anticoagulation effect [7]. More heparin injections are required under these conditions to achieve desired activated clotting time (ACT) values, which may result in bleeding. The situation is significantly worse in newborns as their AT level is lower than that of adults [18].

Heparin use is associated with immune-mediated side effects known HIT and specified by thrombocytopenia and a paradoxical prothrombotic state in heparin treatment. While it has been reported that HIT is a rare phenomenon in neonates [19, 20], it occurs in 0.8–7% of the adult ECMO patients [21, 22]. It has been demonstrated that 30–60% of HIT patients experience thrombotic complications [23, 24]. Therefore, in order to address the aforementioned issues, it is necessary to provide alternative anticoagulants to heparin, such as bivalirudin [20].

### Bivalirudin

DTIs, unlike UFH, do not rely on AT to act as an anticoagulant, but instead they directly inhibit both free circulating and fibrin-bound thrombin. Bivalirudin is a reversible thrombin-binding synthetic bivalent analogue of hirudin with excellent pharmacological profiles [25]. Since bivalirudin can inhibit plasma thrombin, clot-bound thrombin, and collagen-induced platelet activation without forming a complex with the cofactor AT III, it has a much higher bioavailability than heparin [26, 27]. It has a short half-life of approximately 25 min in patients with normal renal function, making it suitable for rapid titration [28, 29]. It is mostly metabolized by the liver through proteolytic cleavage, but it also partially cleared by the kidney (20%), so the dose should be adjusted during renal dysfunction as it prolongs its half-life [30, 31]. Bivalirudin has been used to prevent clotting during ECMO in both HIT patients and non-HIT patients [32–34]. It is administered intravenously in doses ranging from 0.025 to 0.48 mg/kg/hour, with an action time of 2–4 min [35]. Bivalirudin efficacy has been shown to correlate well with both ACT and aPTT results [36, 37]. The researchers also compared the bivalirudin and UHF aPTT results and discovered that using bivalirudin yields more stable aPTT results [34, 38]. There is no unified approach for bivalirudin infusion, so it can be used with or without an initial bolus of bivalirudin with initial loading ranging from 0.04 to 2.5 mg/kg followed by continuous infusion [34, 39]. In particular, Koster et al. used bivalirudin for HIT patients with a bolus of 0.5 mg/kg followed by a continuous infusion of 0.5 mg/kg/h to maintain an ACT of 200–220 s [33]. In another study, Jyoti et al. [39], were able to achieve a target ACT of 200–220 s with an injection rate of 0.1–0.2 mg/kg/h and no bolus dosage of bivalirudin. The dose of bivalirudin is maintained at 0.03–0.2 mg/kg/h to maintain therapeutic targets, with [19, 32, 33] or without [34] an additional initial amount of 0.5 mg/kg. In a meta-analysis, it has been reported that in-hospital mortality, major bleeding events and pump-related thrombosis were less frequent in DTI compared to heparin [40].

There are some considerations before bivalirudin usage in ECMO. First, it affects the renal clearance process in patients with impaired renal function, resulting in drug accumulation. Therefore, lower dosage of bivalirudin is required for patients with renal dysfunction [41]. Moreover, since bivalirudin is rapidly metabolized where the blood is in stasis, it is not a viable option in venoarterial ECMO [42]. Another limitation of the bivalirudin is that there is no antidote in case of overdose or bleeding, which makes bleeding management challenging. Bivalirudin resistance may exist in the absence of a clear etiology [43]. APTT, ECT, and plasma-diluted thrombin time tests are typically used for monitoring anticoagulant effect of bivalirudin [44, 45].

### Argatroban

Argatroban is a synthetic direct thrombin inhibitor with a half-life of 39–51 min [46] and is not recommended for patients with severe hepatic dysfunction since it is metabolized in the liver, therefore, renal failure is not a concern. One of the primary issues in ECLS that limits DTIs adoption is a lack of pharmacologic antidote. However, due to their short half-lives, if bleeding occurs, the injection of DTIs can be stopped or reduced to stop the bleeding. Argatroban has been utilised as an alternative to UHF in cases of suspected HIT in adults, pediatrics, and neonates on ECMO. Its maintenance dose is 0.1–0.65 $\mathrm{\mu g}/\mathrm{kg}/\mathrm{min}$ [47, 48], and centres use a 100–200 $\mathrm{\mu g}/\mathrm{kg}$ initial bolus dose [35].

### Fondaparinux

Fondaparinux is a factor Xa inhibitor that has been indicated to be effective as an anticoagulant agent in severe acute HIT [49]. Parlar et al. [50] used fondaparinux daily subcutaneous injections (2.5 mg per day) in ECMO loop for a patient with HIT and found no adverse effects. Compared with DTIs which require the aPTT or the ECT monitoring methods, anti-FXa assays are more reliable for fondaparinux monitoring since anti-FXa assays do not rely on patient factors. To delineate, the aPTT and the ECT are influenced by the prothrombin level of the HIT patients which is often low and results in falsely long aPTT and, consequently, in inappropriate dose of the anticoagulant [51].

### Nafamostat mesylate

Nafamostat mesylate is a synthetic serine‐protease inhibitor that inhibits many procoagulant factors, including thrombin, plasmin, trypsin, kallikrein, factors XIIa and Xa [52]. In the literature, there are conflicting findings for the use of NM in ECMO. Lim et al. [53] investigated thromboembolic or bleeding complications during ECMO using heparin and NM. According to their findings, bleeding complications were more common in patients receiving NM, while thromboembolic problems were comparable in both cases. Other studies, on the other hand, claim that it is an appropriate alternative to heparin that reduces the risk of bleeding in ECMO patients [54, 55]. Like other anticoagulants, there is no unified approach for the dosage rate but typically it falls into the range of 0.26–0.93 mg/kg/hr [54–56].

### Warfarin

Warfarin is an oral anticoagulant that inhibits the utilisation of vitamin K (factors II, VII, IX, and X). The main advantage of this method is its ease of administration and reversibility [57]. When patients have been adequately anticoagulated with DTIs and require long-term anticoagulation after the acute period of HIT, they are typically switched to vitamin K antagonists (VKA) such as warfarin and phenprocoumon [58]. Warfarin dosage should be determined based on the patient's response to the drug, and it can be monitored using international normalised ratio (INR) analysis to keep its results in therapeutic range [2, 3, 59]. Lee et al. describe the successful use of ECMO as a bridge-to-recovery therapy in a patient suffering from fatal warfarin-exacerbated DAH [60].

### Anticoagulation monitoring methods

Given the importance of anticoagulant monitoring and dose adjustment, it is vital to determine the appropriate approach for each anticoagulant and its dosage. Partial thromboplastin time (aPTT), anti-factor Xa assay, D-dimer, PT/INR, Full blood counts (FBC), Fibrinogen, ECT, activated clotting time (ACT), and viscoelastic tests (ROTEM/TEG) are discussed in this section. Also, recently developed novel real-time monitoring methods including sound, optical, fluorescent, and electrical measurements methods are presented. Table 2 summarises the sample type, purpose, target range, advantages, and disadvantages of different techniques.

**Table 2:** Conventional anticoagulation monitoring methods

Method	Sample type	Advantages	Disadvantages	Purpose	Desired range	Ref
aPTT	Plasma	● Widely available ● Well known method ● Easy to interpret ● Not affected by platelet count	● Nonspecific to heparin ● Time-consuming and user-dependent ● Can be affected by various parameters, such as drugs, hematocrit, abnormalities in coagulation factors, fibrinogen, high C-reactive protein ● Extended clotting time in the presence of lupus anticoagulant	To monitor anticoagulant effect	25–90 s or 1.5–2.5 times baseline (depending on the device, anticoagulant, and utilized method)	[70]
Anti-factor Xa assay	Plasma	● Sensitive to UFH ● Independent of coagulopathy, thrombocytopenia, or dilution ● Correlates better than ACT and aPTT with heparin concentration	● Affected by hyperlipidemia and hyperbilirubinemia, haemolysis, lipaemia, AT, and high plasma-free haemoglobin ● Not easily accessible ● Costly ● Time-consuming ● Only measures inhibition while not showing the amount of fibrin and thrombin generated	To monitor anticoagulant effect	0.3–0.7 IU/mL	[71]
ACT	Whole blood	● Point-of-care test ● Low cost and fast ● Simple operation ● Better correlate with high concentrations of heparin Small sample volume	● User and instrument dependent results ● Lack of specificity Sensitive to various parameters including platelet dysfunction, platelet inhibitors (e.g., GP IIb/IIIa), temperature, haematocrit, blood thrombocyte activity, haemodilution, anemia, hypothermia, coagulation factors deficiencies, hypofibrinogenemia, fibrinogen, thrombocytopenia, AT level, oral coagulants, and activator type	To assess anticoagulant effect	180–220 s	[72]
D-dimer	Whole blood or plasma	● Prognostic value for oxygenator failure ● High sensitivity	● Time-consuming ● Relatively expensive ● Moderate specificity	To monitor fibrin formation and fibrinolysis in the circuit and patient’s body	0.28–1 mg/L	[73]
PT/INR	Plasma	● Indicator of various factors including factors I, II, V, VII, and X	● Interfere with DTIs ● Deficits in common factors I, II, V, and X can prolong PT	To assess underlying coagulability and to monitor Vitamin K antagonists	< 1.5 for heparin-treated patients, > 2–3 for other anticoagulants	[74]
ECT	Plasma	● Simple operation ● Not sensitive to heparin	● Rely on both prothrombin and fibrinogen ● Lack of standardization and uniformity ● Results affected by DTIs	To monitor the effect of DTIs	300–500 s	[75]
Viscoelastic tests (ROTEM/TEG)	Whole blood	● Point-of-care test ● Distinguishing clotting factor deficiency, platelet dysfunction, and hyperfibrinolysis	● Poor specificity ● Low sensitivity to hyperfibrinolysis ● Sensitive to vibration ● Extended clotting time in the presence of lupus anticoagulant ● Lack of standardization and uniformity	To assess clotting time (anticoagulant effect), thrombocytopenia, hypofibrinogenemia, and fibrinolysis	Not established	

*FBC* Full Blood Count, *aPTT* Partial Thromboplastin Time, *PT/INR* Prothrombin time/international normalized ratio, *DTI* Direct thrombin inhibitors, *ECT* Ecarin Clotting Time, *ACT* Activated Clotting Time, *ROTEM* Rotational Thromboelastometry, *TEG* Thromboelastography), *UFH* Unfractionated heparin

### Activated partial thromboplastin time

APTT is an anticoagulant monitoring technique that is most commonly used to assess the effect of heparin and bivalirudin [76]. It is defined as the time required for calcium-free plasma to generate clots after being exposed to fibrin-activating reagents and calcium. Clot formation can be detected using a variety of analytical methods, including optical, mechanical, and electrochemical techniques [77]. This method involves combining citrated plasma, a phospholipid, calcium, and a contact pathway activator (silica, celite, kaolin, ellagic acid, polyphenolic acid) to trigger clot formation [78]. The normal range is defined in most laboratories as 25–90 s (an aPTT level of 1.5–2.5 times baseline is recommended for anticoagulation monitoring) but it varies from clinic to clinic and is determined by the instrument and reagents used and it is critical not to extrapolate data from one ECMO centre to another without knowing the method and assay used [79, 80]. Bates et al. investigated the relationship between aPTT and anti-factor Xa assay heparin level using four different automated coagulometers and six commercial aPTT reagents. Their findings revealed that, while there is a good correlation (*r = *0.64 to 0.95) between aPTT anti-factor Xa assay results, the aPTT values at 0.3 IU/mL plasma heparin concentration determined by anti-factor Xa assay will range from 48 to 108 s, depending on the instrument and reagent utilised [81].

Another concern with this method is that it can be influenced by parameters, including drugs, hematocrit, acute phase reactants, abnormalities in coagulation factors, high C-reactive protein, hyperbilirubinemia, hyperlipidemia and lupus anticoagulant [82] so that deficiencies in common pathway factors I, II, V, and X, as well as contact pathway components such as high-molecular-weight kininogen, prekallikrein, and factors VIII, IX, XI, and XII, and lupus anticoagulants can prolong the aPTT results [78].

### Anti-factor Xa assay

Anti-factor Xa is a functional chromogenic assay for coagulation monitoring and evaluating the effective anticoagulant concentration. The anti-Xa assay is specific to heparinoid’s action and is unaffected by deficits in other coagulation factors and can be used with or without exogenous AT. In the former method, the sample is treated with sufficient AT, so that the rate-limiting reagent, which is heparin, can inhibit Xa and produce a precise measurement of heparin in the patient sample. In this method, a specific amount of coagulation factor Xa conjugated with chromophore and AT is added to patient plasma containing heparin [83]. Following that, as a result of the chromogenic reaction, AT and heparin form an inhibitory complex that inactivates factor Xa. The activated factor X is then introduced to the sample, which cleaves the chromophore compound, and the amount of released chromophore is measured using spectroscopy. As the amount of remaining factor Xa in the sample is inversely proportional to the original amount of heparin, the colour change will be greater as less heparin/AT complex interacts with factor Xa, indicating a lower drug level. The relationship between AT and heparin is critical because, even with a high level of heparin, a deficiency in AT causes more unbound factor Xa, implying lower heparin levels. On the other hand, kits without AT do not add extra AT and give a more precise measurement of in-vivo anticoagulation because the patient's AT and heparin levels are both rate-limiting reagents. However, this approach has the problem of being unable to differentiate between AT deficiency and inadequate heparin [84, 85].

The widely accepted target range for anti-factor Xa levels during ECMO is 0.3–0.7 IU/mL [86, 87]. Unlike the ACT and aPTT methods, this method is unaffected by coagulopathy, thrombocytopenia, or dilution and best represents the overall heparin anticoagulation level. However, some parameters which can occur in patients on ECMO, such as hyperbilirubinemia, haemolysis, lipaemia, hyperlipidemia, and plasma-free haemoglobin affect anti-Xa assay results [88, 89]. Anti-Xa activity was observed to be significantly lower in ECMO samples with plasma free haemoglobin levels of 50 mg/dL or above when compared to normal samples: 0.33 (0.25–0.42) versus 0.4 (0.31–0.48) IU/mL [86, 87, 90]. It was stated that anti-Xa assay correlates better with heparin concentration than ACT and aPTT [91–93]. Nankervis et al. studied 12 neonates on ECMO to determine the appropriate heparin dosage based on ACT and anti-Xa results. Their findings show a strong correlation between anti-Xa test and heparin dosage (*r = *0.75; *p* < 0.0001), however ACT results do not correlate with either anti-Xa or heparin dosage [94]. Also, in a cohort study, Bembea et al. [95] discovered a moderate correlation between anti-factor Xa results and heparin injection dosage (*r = *0.33) in 34 extracorporeal life support (ECLS) pediatric patients, but a poor correlation with ACT (*r = *0.02) and aPTT (*r = *0.17) for all patients. It is worth noting that patients on ECMO who are being monitored by anti-Xa levels compared to ACT have fewer blood draws for monitoring, a longer duration between circuit changes, and lower transfusions and dosages of activated factor VII [93, 96].

While anti-factor Xa assay can measure the heparin effect, it cannot reflect other coagulation parameters or the patient's overall hemostatic condition [97]. In fact, the anti-Xa method only measures inhibition, not the amount of fibrin and thrombin produced in the patient's body. Moreover, compared with ACT and aPTT, anti-factor Xa assay more expensive and not easily accessible in all laboratories and hospitals and its results are affected by hypertriglyceridemia (triglyceride level > 360 mg/dL) and hyperbilirubinemia (bilirubin level > 6.6 mg/dL) [98].

### Activated clotting time

ACT is the widely used and well-established point-of-care (POC) method for anticoagulation monitoring. In this method, whole blood is transferred to a tube coated with various activators, such as glass, celite or kaoline, ellagic acid, diatomaceous earth, silica, calcium, and phospholipids, to stimulate the contact activation pathway and the coagulation response (fibrin clot formation) is measured over time in seconds. The mobility of a magnet during clot formation, or the variations in the velocity of blood movement as it begins to clot, is measured and recorded over time. When compared to laboratory-based methods, the advantage of ACT is that it can be conducted as a whole-blood test on a bedside machine, requires a little sample volume, is low-cost, and can be done by unacquainted individuals [99]. The amount of sample required for testing varies between 10 $\mathrm{\mu L}$ and 2 mL depending on the ACT machine. However, several factors including hemodilution, platelet dysfunction, hypothermia, anemia, hypofibrinogenemia, thrombocytopenia, platelet inhibitors (e.g., GP IIb/IIIa), and coagulation factor deficiencies can affect ACT results [100, 101].

There are currently no standardized target range for ACT, however the range of 140–240 s are commonly used in clinical practice [7, 102]. The aPTT approach works well for Unfractionated heparin (UFH) values between 0.1 and 1 U/mL, but the ACT method works better for UFH concentrations between 1 and 5 U/mL. Therefore, based on the heparin level used in ECMO, ACT shows a poor correlation with heparin concentration, while aPTT yields acceptable results in neonates and adults [103–105]. Several studies have been conducted to investigate the relationship between ACT, aPTT, and anti-Xa activity [106, 107]. Khaja et al. discovered that in neonates, aPTT has a stronger correlation with anti-XA than ACT [105]. Although ACT is a good indicator for high heparin concentrations in cardiopulmonary bypass (CPB), low-range ACT (ACT-LR) was proposed for use in the lower heparin dosage range (150–200 IU/kg) used in ECMO [61, 105]. It is worth noting that most hospitals use ACT or ACT-LR methods for routine coagulation monitoring in ECMO.

### D-dimer

The D-dimer protein is the cleaved product of the fibrinolysis process. Therefore, the presence of D-dimers in the blood signals clot lysis. D-dimer levels are higher in patients with disseminated intravascular coagulation (DIC) caused by sepsis, deep vein thrombosis (DVT), pulmonary embolism, or other thrombotic disorders. The D-dimer level, which describes the cross-linked fibrin degradation products generated by reactive fibrinolysis, has also been shown to be a marker for determining oxygenator functionality and tracking oxygenator malfunction [108]. In a retrospective study it was found that D-dimer level increased significantly within 3 days before exchange from 15 [9–20] to 30 [21–35] mg/dL (*P* = 0.002) and declined significantly within 1 day thereafter to 13 [7–17] mg/dL (*P =* 0.003) [109]. Although the oxygen transferred rate is commonly used to calculate the exchange time for the oxygenator, the D-dimer level, and particularly a steadily increasing level in the absence of an alternative explanation, is an alternative method for calculating the appropriate time for membrane exchange.

### Prothrombin time and international normalized ratio

Prothrombin time (PT) is one of the main clinical coagulation monitoring methods. Prothrombin is a protein made by the liver that aids in blood clotting formation. This method involves adding thromboplastin (a mixture of tissue factor, calcium, and phospholipid) to patient plasma and measuring the time it takes for the plasma to clot [110]. The PT results are sensitive to thromboplastin components so using different thromboplastin reagents results in different Prothrombin times, even with the same plasma sample. Therefore, to have consistent and unified results, the World Health Organization (WHO) proposed the international normalized ratio (INR), which has since become the standard method for PT reporting [111]. In this way, INR represents the ratio of PT divided by a control PT value determined by using a WHO-developed international reference thromboplastin reagent. The target INR for a heparin-treated patient should be less than 1.5. Researchers also discovered that the PT/INR value for other anticoagulants including VKA (e.g., warfarin), DTIs, or the oral Factor Xa inhibitors, is in the range of 2–3 or higher [112]. DTIs interfere with the prothrombin time and deficits in common factors I, II, V, and X can prolong PT results [113]. INR was developed to account for discrepancies in laboratory PT reagents and to standardise vitamin K antagonists (VKA), such as warfarin therapy monitoring [78].

### Ecarin clotting time

Ecarin is a metalloprotease isolated from the venom of a saw-scaled viper echis carinatus and a specific activator of prothrombin that can be used to measure the activity of DTIs, such as argatroban and dabigatran [114, 115]. In this method, clotting time is measured after the addition of ecarin solution to diluted plasma. The conversion of fibrinogen to fibrin is used to measure the proteolytic activity of non-inhibited meizothrombin. ECT was successfully implemented by Teruya et al. for bivalirudin concentration monitoring in ECMO [62]. Although this method is not widely used in ECMO, it has been demonstrated that ECT results have a strong correlation with bivalirudin concentration [37]. Furthermore, Alouidor et al. recently proposed a point-of-care ECT analysis device with a good correlation with bivalirudin and dabigatran concentrations that only requires 5 $\mathrm{\mu L}$ of whole blood [116]. Finally, it seems that more research into this approach in ECMO settings with various anticoagulants is required.

### Viscoelastic tests

Thromboelastography (TEG) and rotational thromboelastometry (ROTEM) are point-of-care viscoelastic tests used in ECMO patients to monitor coagulation in the presence of a various stimulating agents [117–119]. Unlike other methods, such as ACT, aPTT, and PT/INR, which only show the endpoint (whether or not a thrombus has formed), this assay can reveal information about different aspects of the coagulation cascade, the clot formation dynamics, clot strength and clot lysis, and fibrinolysis [120–122]. The dynamic changes in the viscoelasticity of whole blood during clotting under low shear stress are recorded. A torsion wire or pin is used to measure the strength of the formed clot. The wire in TEG is stationary and the cup oscillates while in ROTEM, the cup is stationary while the pin oscillates [123]. The oscillations are hampered as the clot forms, and both the TEG and ROTEM devices detect and convert changes in oscillations into the numerous measurements evaluated. In this method, a pair of samples with and without heparinase addition are tested to investigate hemostasis in the presence of UFH [124]. The effectiveness of UFH is then determined by comparing the clotting time in both samples, which is a good indicator in cases where heparin resistance is a concern. The analysis duration for different parameters varies, so coagulation factors can be assessed in 5 min, and fibrinolysis analysis takes 60–90 min. While viscoelastic tests provide useful information about the coagulation system, it does not provide data on the level of von Willebrand factor (VWF), which is a good indicator of potential bleeding or hemostasis condition [125]. Although the use of viscoelastic tests in ECMO patients has increased in recent years, it is still not widely available in most centres [126] which can be due to a lack of generalised therapeutic ranges in the neonatal population [17].

### Novel real-time anticoagulation monitoring methods

This section presents recently developed novel real-time monitoring methods such as sound, optical, fluorescent, and electrical measurements. Furthermore, a brief explanation of each approach and its application in ECMO circuits is provided.

### Sound monitoring method

**Fig. 3:**
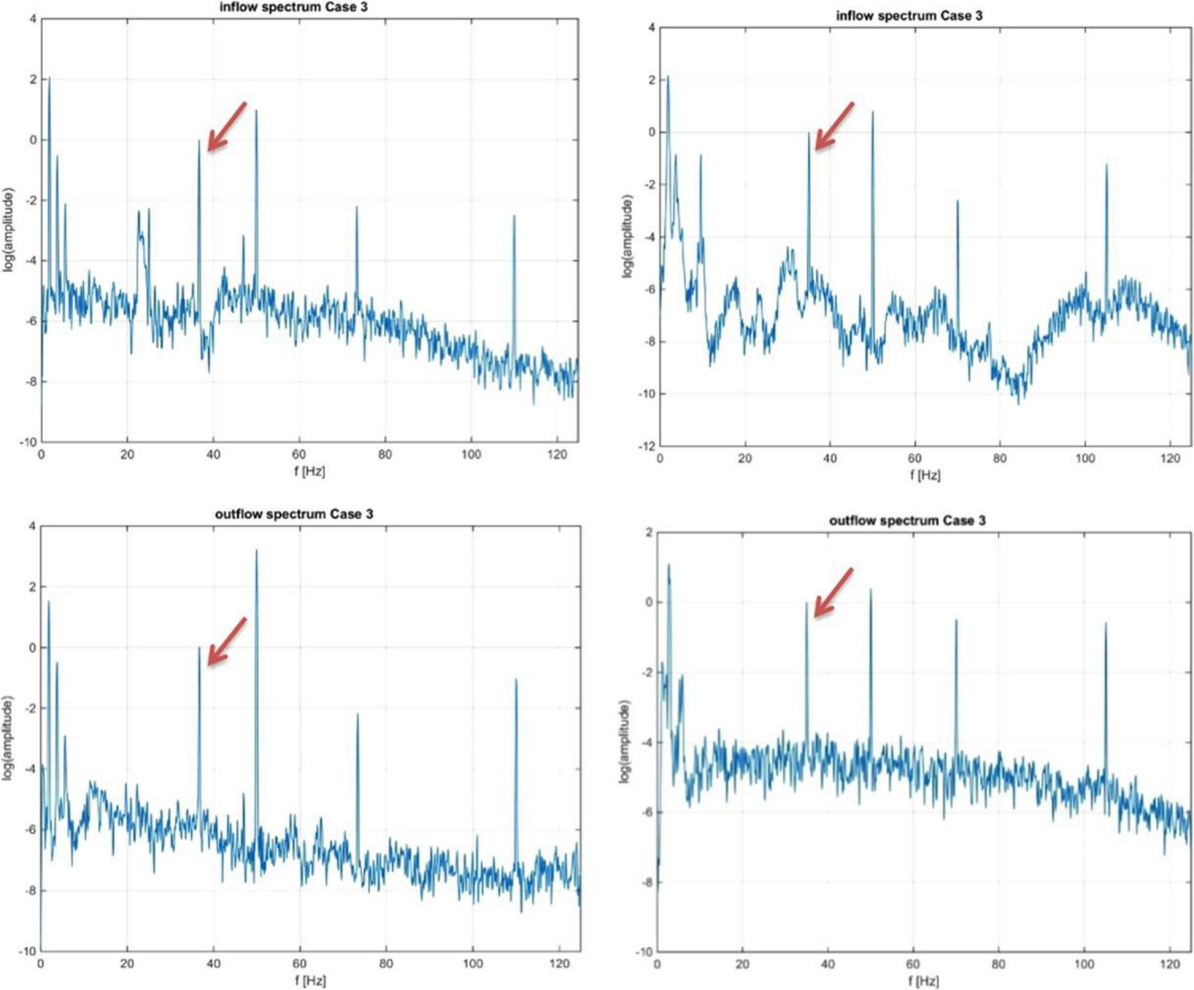
Acoustic spectrum of an infant patient in ECMO, the upper-left and lower-left spectra exhibit the inlet and outlet of a pump associated with an infant patient, respectively, while the upper-right and lower-right spectra depict the inflow and outflow spectrum, respectively, after 5 days for the same patient. Reused with permission from Ref. [127]

Due to the high shear rates in the ECMO pump, it is one of the suspect points for clot formation. Measuring the sound generated by the pump is a promising method for detecting clot formation in the pump and preventing potential patient and pump damage. Fuchs et al. [127] discovered a link between sound signals and clots in the pump's inlet and outlet while using ECMO. The frequency spectrum changes while blood clots move through the pump at different pump speeds, which is a reliable indicator of clot formation inside the pump (Fig. 3). It has been illustrated that the acoustic analysis method is a low-cost, simple, and easy-to-access method that can provide reliable results and detect thrombosis formation in the pump in the early stages of thrombosis formation. Although this is a non-invasive technology, it can only be used to monitor thrombosis in the pump because the analysis is based on the sound made by the pump [128].

### Optical method

**Fig. 4:**
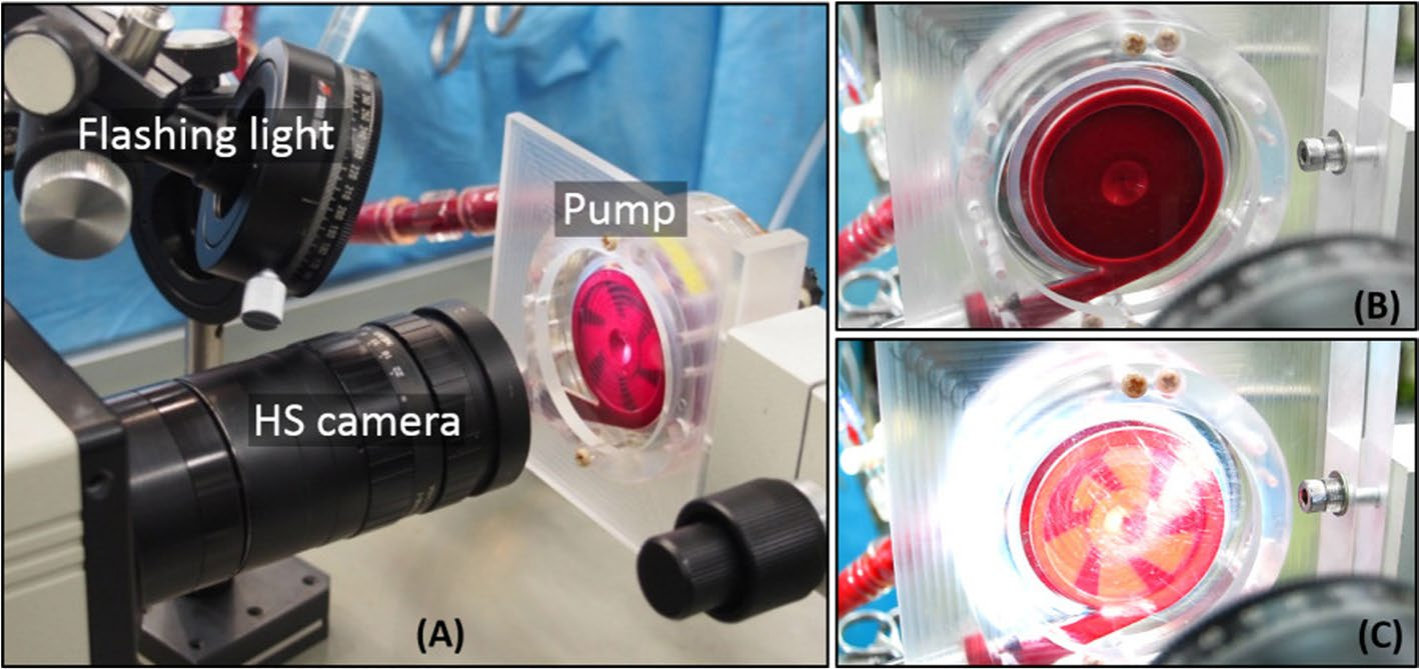
**a** Illumination of the bottom surface of the pump by using stroboscope; Using the stroboscope method, it is feasible to view high-speed rotation of the impeller **c** which is not achievable without the use of the stroboscope method **b** Reused with permission from Ref. [130]

Optical methods are non-invasive and real-time thrombus monitoring techniques that detect the light intensity scattered by blood at different wavelengths [129]. Fujiwara et al. [130] utilized a hyperspectral imaging (HSI) technique to visualize thrombosis formation within a levitated centrifugal pump, which is commonly used in ECMO devices (Fig. 4). Two groups of pigs were employed in this study undergoing ECMO and LVAD. The purpose of this study was to detect thrombosis inside the centrifugal ECMO pump and the source of the thrombosis. In this way, multiple real-time images of the inside of the pump were acquired with the HS camera over the wavelength range of 608–752 nm. Within 24 h of blood circulation, thrombosis was discovered, arising from both the inside and outside of the pump. Thrombosis generated outside the pump was identified to form around the inlet cannula and junction between the pump inlet and tubing. It is worth noting that optical methods have some limitations. First, the installation and adjustment of the distance for hyperspectral camera focusing should be considered. Furthermore, due to the limitations on bending radius, optical fibres are fragile.

In another study, Morita et al. [129] used a micro-optical monitoring method with an extracorporeal circuit to detect thrombus in fresh porcine blood. Their setup composed of two micro-optical thrombus sensors which detect scattered light at two wavelengths, 660 and 855 nm. One optical sensor was placed on the rotary pump (to monitor clotting) and the other on the flow channel (less suspicious thrombus formation point, as reference point). In this vein, each LED emits 660 and 855 nm light into the blood flow, which is absorbed or scattered by blood components (most notably red blood cells (RBCs)) and then detected by a photodiode. To monitor thrombus formation in the pump, the ratio of light intensity at each wavelength for both sensors was eventually measured. They also depicted that the proposed micro-optical sensor has no installation limitations and allows researchers to install additional micro-sensors at various points where thrombus formation is suspected.

### Fluorescence method

**Fig. 5:**
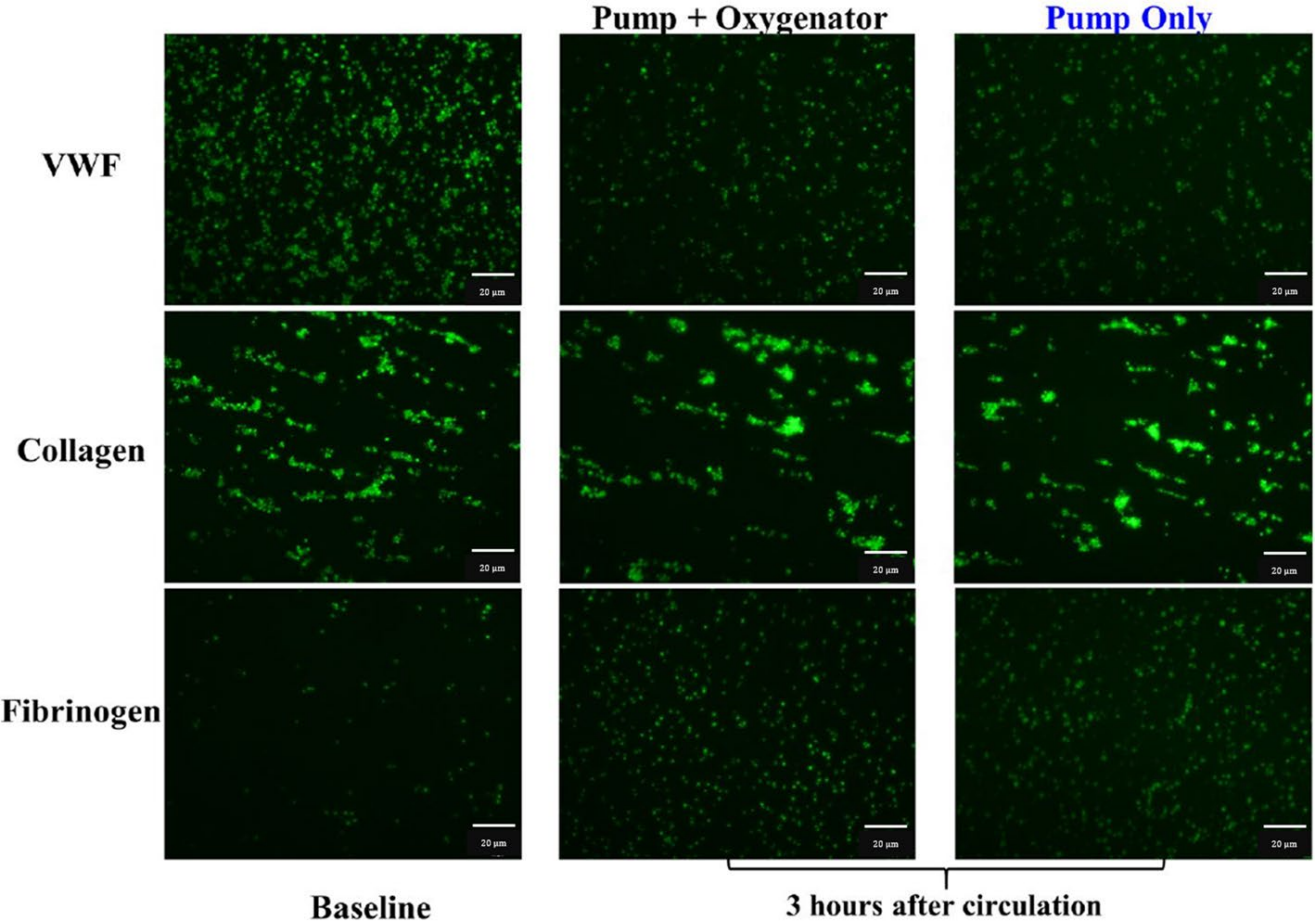
Fluorescent images of adherent platelets on VWF, collagen, and fibrinogen of the blood samples at baseline and three hours after circulation in the two circuits. Modified and reused with permission from Ref. [132]

Fluorescence microscopy is another monitoring method for thrombin formation, which measures the onset of thrombus formation, which produces fibrin in the blood. By fluorescently labelling fibrinogen, it is possible to evaluate the microscopic clot formation process [131]. Therefore, the clotting time can be calculated by measuring the variation and distribution of fluorescent intensity over time and, consequently, the influence of heparin concentration on blood clotting time. Considering that high shear rate regions are more suspect for clot formation, Sun et al. [132] utilized a whole blood flow cytometry assay to measure platelet activation in a fresh human blood sample in the centrifugal pump and oxygenator in the ECMO circuit. It was found that platelet activation and adhesion on fibrinogen increases after 4 h of running ECMO which can result in thrombosis formation. On the other hand, GPIbα and GPVI platelet receptors population decreases over time weakening platelet adhesion to collagen and VWF, resulting in bleeding complications (Fig. 5).

### Electrical measurement

**Fig. 6:**
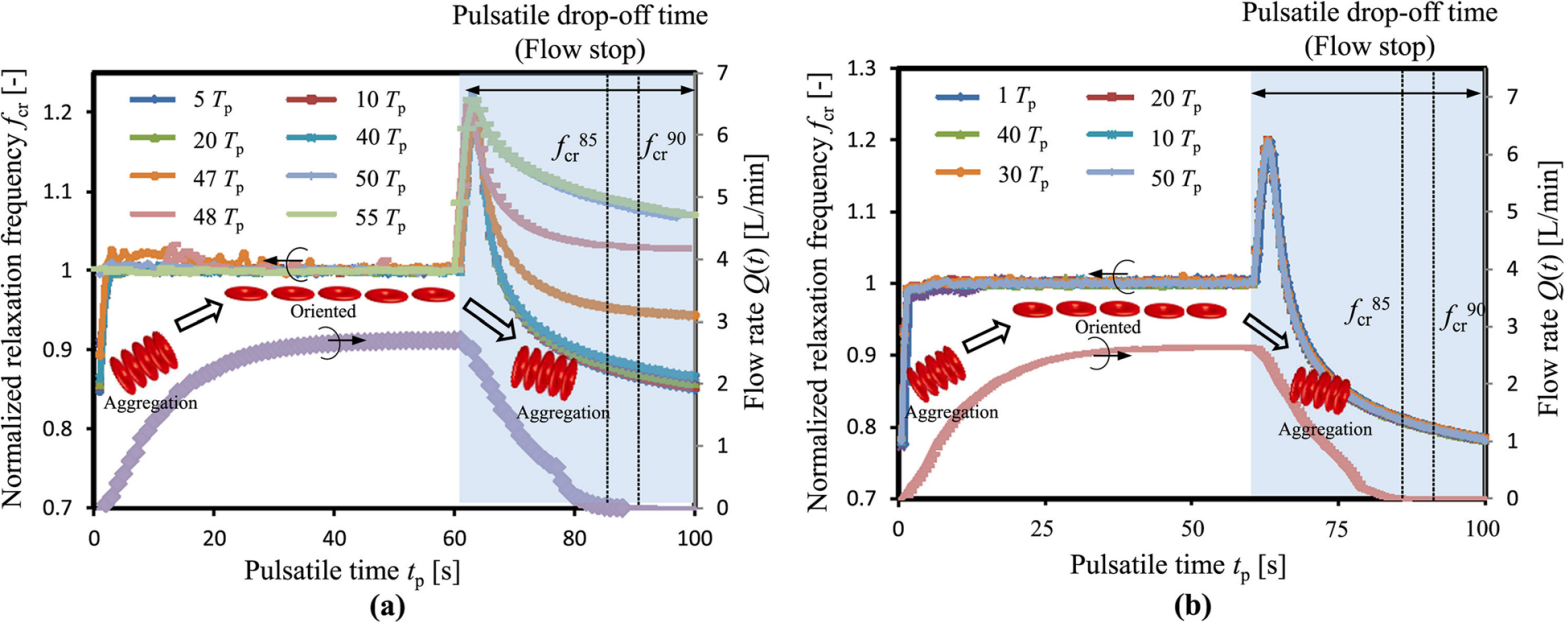
Experimental results illustration: **a** Porcine blood with coagulation, **b** Porcine blood without coagulation. Reused with permission from Ref. [134]

Electrical impedance measurements can be used to monitor blood coagulation in ECMO systems. Based on single or multiple-frequency electrical impedance measurements, it has been demonstrated that there is a relationship between thrombus formation and electrical resistivity/permittivity [133]. Red blood cells (RBCs) have phospholipid on their surfaces, they can activate factor IX causing coagulation. Therefore, it can be deduced that RBC aggregability is related to thrombus formation. Using multiple-frequency electrical impedance spectroscopy, Li et al. [134] investigated RBC aggregability in an extracorporeal circulation system with pulsatile flow. Their results revealed that in coagulating blood, RBCs aggregability decreases, indicating thrombus formation. ACT and fibrinogen were examined to assess the relationship between aggregability and blood coagulation, and their results show a decrease in their level over time, similar to RBC aggregability (Fig. 6). It is also worth noting that electrical impedance measurement can be readily applied to low-cost, compact, and simple POC blood coagulation testing devices. However, the limitation of this method is that it is invasive because the electrodes must come into contact with the blood.

### Future perspectives

It is essential to find appropriate anticoagulants and related monitoring methods to balance between thrombosis and bleeding with ECMO. Factor XII inhibitors are promising anticoagulant agents which are currently under investigation in in-vivo ECMO models. It has been demonstrated that coating the surface with FXIIa‐directed corn trypsin inhibitor prevents blood clotting when catheters are inserted into rabbit jugular veins [135]. Also, it has been illustrated that knocking out FXII enhances the time to catheter occlusion. Recently, researchers found that human antibody 3F7 binds to FXIIa, preventing blood clotting within the ECMO circuit [136]. Antibodies have an advantage over other anticoagulants in that they do not increase the risk of bleeding. Although it has only been tested in animal ECMO circuits, FXII-directed therapy appears to be a promising method for clot prevention on ECMO support [137].

It has recently been demonstrated in a variety of clinical settings that using low-dose anticoagulation is an effective strategy to decrease bleeding complications and enhance survival rate while patients are receiving ECMO, as opposed to when a standard dosage is used [138, 139]. Although thrombosis is still a concern, recent studies suggest that there is no difference in thrombosis complications and hospital mortality between low-dose and therapeutic anticoagulant dosage [140]. While several studies have now demonstrated the benefits of using low-dose anticoagulation in adults to reduce bleeding while still preventing major thrombosis, there is still a lack of research on the efficacy and safety of using low-dose anticoagulation in pediatric patients undergoing ECMO. As a result, extensive clinical research and trials are required to determine the optimal low anticoagulant dosage in both adults and pediatric patients.

In another approach, shear-sensitive drug delivery systems may protect the ECMO circuit from coagulation and thrombosis formation by modifying commonly used generalized drug delivery which results in bleeding complications. Localized drug delivery method is a novel approach which relies on material deformation or disaggregation to elicit drug release [141]. Nanoparticles can be used for nanotherapeutic targeting drug delivery systems that, like platelets, can activate (rupture) under high shear stress and release the drug at the site of action to prevent blood clotting [142]. This characteristic enables the development of shear-sensitive nanoparticles capable of releasing antithrombosis drugs only at high shear stress points, such as the cannulas, pump and oxygenator while remaining intact at lower shear rates. This is beneficial because it not only reduces the amount of anticoagulant used but also aids in localized drug delivery, which only releases the drug where it is required, potentially resulting in less bleeding.

Recently, an anticoagulant-free method based on surface modification of the ECMO circuit was developed to decrease bleeding and thrombosis complications [143]. This method is based on the application of appropriate surface coating techniques to make the surface of the tubing, membrane oxygenator, and pump biocompatible and avoid thrombosis formation. Several innovative approaches, such as polymer coating, nitric oxide (NO) coating, and tethered liquid perfluorocarbon coating, are presented that can improve ECMO hemocompatibility. When considering surface coating technologies, it is also crucial to consider gas exchange rate, pressure drop, and shear stress inside the membrane oxygenator. These features must be incorporated to increase the stability and durability of the materials suitable for biocompatible surface coatings.

Various real-time analysis methods for thrombosis monitoring are presented in this review. Although these techniques do not often show the coagulation status quantitatively, there are low-cost, compact, and user-friendly POC equipment available that can be used at the patient's bedside. POC diagnostics are promising techniques for evaluating anticoagulation impact when compared to conventional standard laboratory-based assays [144]. Laboratory-based testing is time-consuming, costly, and user-dependent. POC devices have several advantages, including reliability, quick response, and reduced sample consumption [145]. Some conventional coagulation monitoring methods, such as d-dimer, aPTT, PT/INR, and ECT, have recently been developed as POC devices [146]. Although the benefits of POC devices are well established, and various POC devices have been introduced, there is always a need to study novel devices to improve the accuracy and reliability of the results. Microfluidic devices, in particular, can be exploited for anticoagulation monitoring due to benefits such as low cost, simple operation, quick analysis, and reduced sample consumption. Furthermore, because microfluidic systems are high throughput, several samples can be analysed simultaneously with a small amount of sample in a single integrated platform [147]. Although microfluidic platforms have been developed and are currently being used in research laboratories, they are rarely marketed. Commercialisation of low-cost and high-throughput microfluidic devices for monitoring the coagulation pathway would provide unique advantages in the clinical management of patients receiving ECMO.

## Conclusion

Although heparin is still the gold standard anticoagulation agent utilized in ECMO, heparin resistance and HIT are major clinical limitations. Next-generation anticoagulants also have their own shortcomings, which may include: short half-life, lack of antidots and restrictive monitoring methods that hamper broad clinical implementation. Several anticoagulant monitoring methods have been developed to track the patient's coagulation condition and adjust the anticoagulant dosage; some monitoring methods correlate better with specific anticoagulant agents than others. To establish an optimal balance of anticoagulation and bleeding requires a variety of approaches, rather than a single monitoring method, to monitor anticoagulation impact. Existing research efforts should be directed towards the development of novel anticoagulants, surface engineering to modify non-biocompatible surfaces, targeted drug delivery systems, and the development of real-time and POC monitoring methods that will result in fewer thrombosis and bleeding complications.

## Data Availability

Not applicable.

## Abbreviations

ACT: Activated clotting time
ACT-LR: Activated clotting time-low range
aPTT: Partial thromboplastin time
AT: Antithrombin
CPB: Cardiopulmonary bypass
DOAC: Direct oral anticoagulant
DTI: Direct thrombin inhibitors
DTT: Dilute thrombin time
ECLS: Extracorporeal life support
ECMO: Extracorporeal membrane oxygenation
ECT: Ecarin clotting time
FFP: Fresh frozen plasma
HIT: Heparin-induced thrombocytopenia
HSI: Hyperspectral imaging
LVAD: Left ventricular assist devices
PCC: Prothrombin complex concentrates
POC: Point-of-care
PT/INR: Prothrombin time/international normalized ratio
RBC: Red blood cell
ROTEM: Rotational thromboelastometry
TEG: Thromboelastography
UFH: Unfractionated heparin
VA-ECMO: Venoarterial extracorporeal membrane oxygenation
VV-ECMO: Venovenous extracorporeal membrane oxygenation
VKA: Vitamin K antagonists
WHO: World health organization
